# Self-compassion, ego-resiliency, coping with stress and the quality of life of parents of children with autism spectrum disorder

**DOI:** 10.7717/peerj.11198

**Published:** 2021-05-03

**Authors:** Anna Pyszkowska, Kamila Wrona

**Affiliations:** Institute of Psychology, University of Silesia, Katowice, Silesia, Poland

**Keywords:** Autism spectrum disorder, Parents of children with autism, Self-compassion, Ego-resiliency, Coping, Quality of life

## Abstract

**Background:**

The literature shows a fairly coherent picture of the types of difficulties parents face. Adaptive both coping styles and resources, such as self-compassion and ego-resiliency, indicated as important predictors of the quality of life among parents of children with autism spectrum disorder. The aim of the study was to determine the links between self-compassion and ego-resiliency, coping with stress and quality of life among parents of children with autism spectrum disorder in a Polish sample (*N* = 76).

**Methods:**

A cross-sectional study was conducted. The CISS, Self-Compassion Scale-Short, Ego-Resiliency Scale, and Quality of Life Questionnaire were used.

**Results:**

Regression analysis was carried out to address the research question. It was confirmed that both resources studied exhibited negative relations with emotion-oriented coping, while ego-resiliency was also positively correlated with task- and avoidance-oriented strategies. The hierarchical multiple regression conducted in three steps indicated that ego-resiliency (18%) and emotion-oriented (14%) were the strongest predictors of quality of life among parents of children with ASD.

**Conclusions:**

The obtained results proved that ego-resiliency and a task-oriented coping strategy were important indicators of the quality of life of parents of children with ASD.

## Introduction

Raising a child diagnosed with autism spectrum disorder (ASD) is a substantial challenge for the parents that is often associated with requirements that are considered to be beyond their psychological, material or social scope. The challenges and needs of parents of individuals with ASD are varied: since there is no one pattern of “a typical child with ASD”, there is no one “typical parent of a child with ASD” (Pisula & Porębowicz-Dorsmann, 2017). Although there is no single pattern, the research showed a fairly coherent picture of the types of difficulties parents face. Adaptive both coping styles and resources, such as self-compassion (Torbet, Proeve & Roberts, 2019; Neff & Faso, 2015) and ego-resiliency (Pastor-Cerezuela et al., 2020; Pastor-Cerezuela et al., 2016), indicated as important predictors of the quality of life among parents with ASD.

Quality of life is significantly lower in families raising children with autism than in families of typically developing children (Vasilopoulou & Nisbet, 2016) or children with other disabilities (Brei, Schwarz & Klein-Tasman, 2015). Based on the current literature, one can assume the presence of a vicious circle that drives negative affect in this group. Stress, anxiety, and depression associated with caring for the child result in the cognitive and emotional fatigue of the parent, which leads to an unstable pattern of relations with the child in the context of both emotional and behavioural reactions.

Lai & Oei (2014) indicated that parents of children with ASD most often use problem-focused coping strategies which is in accordance with similar reports regarding task-oriented coping (Dąbrowska & Pisula, 2010). On the other hand, vast research show tendencies towards avoidance-oriented coping strategies in this group (Lai et al., 2015; Pisula & Kossakowska, 2010). Meta-analysis conducted by Vernhet et al. (2019) revealed that problem-focused coping strategies decreased stress levels experienced by parents of children with ASD, while emotion-oriented strategies acted conversely being associated with more stress and depressive symptoms. As reported by Cappe et al. (2011), problem-focused coping strategy exhibited positive relations with quality of life of parents of children with ASD, while emotion-oriented coping strategy demonstrated negative correlations.

Vernhet et al. (2019) pointed out the lack of research regarding links between positive or adaptive resources in this group which limits the applicability of most of the current findings in terms of the improvement of parents’ quality of life and adaptive coping. The results of several resources-focused studies indicated the particular importance of two resources in coping and quality of life in people with chronic stress, including parents of children with ASD: self-compassion and ego-resiliency.

Self-compassion is a term introduced by Neff (2003) that describes compassion towards the self that is not aimed at assessing one’s value, referring to a healthy form of self-acceptance. In assessments of the quality of life of parents of children with ASD, it was demonstrated that self-compassion was a significant predictor of psychological well-being (Torbet, Proeve & Roberts, 2019; Robinson et al., 2017).

Ego-resiliency is a relatively fixed ability of a person to adjust despite objectively difficult environmental conditions (Block & Block, 1980) and is related to the ability to restore balance after traumatic events and effective functioning despite objectively difficult experiences (Masten & Powell, 2003). It has been indicated that among parents of children with disabilities, resilience is an important resource that allowed for more effective coping (Heiman, 2002) and decreased depressive symptoms (Bitsika, Sharpley & Bell, 2013).

The main aim of this study is to identify the relationships between coping strategies, self-compassion, ego-resiliency and quality of life in parents of children with autism spectrum disorder. It was assumed that self-compassion (Lloyd et al., 2019; Allen & Leary, 2010) and ego-resiliency (Vulpe & Dafinoiu, 2012) would exhibit negative relationships with emotion-oriented coping strategies and positive with task-oriented strategies, as well as act as significant predictors of quality of life.

## Materials and Methods

This study was approved by the Ethics Committee of University of Silesia in Katowice (approval no.: KEUS.22/04.2020). All participants provided written informed consent prior to enrolment in the study.

Nonprobability sampling was used. The parents of children with diagnosed autism spectrum disorder (both autism and Asperger syndrome, F84.0) who were benefiting from the help of psychological counselling centre in Bytom, Silesia, Poland, were invited to the study. Children with any additional disorders (for example, epilepsy or physical disability) were excluded. The study was anonymous and voluntary, and the participants did not receive any compensation.

Seventy-six persons participated in the study, including 58 women (M_age_ = 37.76; SD_age_ = 6.54). In total, 42.1% of the respondents lived in cities with more than 100,000 inhabitants. A total of 48.68% were not working, 40.79% worked full time, and 10.53% worked part time.

To measure the variables, socio-demographic metrics and four questionnaires were used. Participants who decided to be involved in the study filled by themselves the following tools:

Coping. To measure coping with stress, the Coping Inventory for Stressful Situations (CISS) by Endler & Parker (1990), adapted into Polish by Strelau, Jaworowska & Szczepaniak (2009), was used. The questionnaire is composed of 48 statements (e.g., “Analyze problem before reacting”) assessed on a 5-point response scale and contains three main scales: task-oriented coping, emotion-oriented coping, and avoidance-oriented coping. CISS’s overall Cronbach’s *α* in the present sample was =.88.

Self-compassion. Self-Compassion Scale Short is a questionnaire composed of 12 statements (e.g., “I try to see my failings as part of the human condition”) aimed at assessing the level of self-compassion of an individual was prepared by Neff et al. Raes et al., 2011. This tool was adapted to the Polish context by Kocur (unpublished data, attached in supplementary material). The Cronbach’s *α* reliability index in the studied group was =.76.

Ego-resiliency. The Ego-Resiliency Scale is composed of 12 statements (e.g., “I get over my anger at someone reasonably quickly”) developed by Block & Kremen (1996) to assess the level of ego-resiliency was used. The Polish adaptation was prepared by Kołodziej-Zaleska & Przybyła-Basista (2018). Scale’s Cronbach’s *α* reliability in the current study was =.81.

Quality of life. A Quality of Life Questionnaire developed by Straś-Romanowska, Oleszkowicz & Frąckowiak (2004) was used; the scale is based on the concept of the multidimensional human being. It is composed of 60 items (e.g., “There are more successes than failures in my life”) for four dimensions of quality of life: psychophysical (satisfaction of fulfilled biological needs), psychosocial (support and the quality of social relationships), subjective (issues of the individuality and independence of a person) and metaphysical (satisfaction from spiritual life). The Cronbach’s alpha reliability coefficient for the whole questionnaire was =.93.

## Results

All data analyses were performed in JASP 0.11.1.

### Coping with stress, ego-resiliency, self-compassion and quality of life in parents of children with ASD

The characteristic of the distribution of the studied variables can be found in Table 1.

**Table 1:** Descriptive Statistics including mean, standard deviations, Shapiro-Wilk estimates.

	Task- oriented	Emotion- oriented	Avoidance- oriented	Engaging in substituting activities	Looking for social contacts	Self- compassion	Ego- resiliency	Psychophysical dimension	Psychosocial dimension	Subjective dimension	Metaphysical dimension	Quality of life
**Valid**	76	76	76	76	76	76	76	76	76	76	76	76
**Missing**	0	0	0	0	0	0	0	0	0	0	0	0
**Mean**	57.90	41.42	39.93	16.95	15.95	36.29	33.30	42.80	45.12	45.95	46.22	180.09
**Std. Deviation**	8.43	10.55	9.57	5.26	4.35	7.32	5.61	6.35	6.06	5.97	5.99	20.61
**Shapiro–Wilk**	0.97	0.98	0.98	0.97	0.98	0.98	0.98	0.99	0.99	0.97	0.96	0.98
**P-value of Shapiro–Wilk**	0.09	0.37	0.17	0.04	0.35	0.27	0.17	0.82	0.75	0.10	0.03	0.34

A Shapiro–Wilk test of normality was conducted. Two of the variables (Engaging in substituting activities and Metaphysical dimension of Quality of life) were found to be significant (*P* < .05). Based on this results non-parametric Spearman correlation was conducted. Results are presented in Table 2.

Task-oriented coping strategy showed moderate positive correlations with every dimension of quality of life. The strongest correlation was with the metaphysical dimension (*r*_*s*_ = .44, 95% *CI* [.239, .606), *P* < .001). In turn, emotion-oriented coping had significant moderate negative correlations with all dimensions of quality of life. The strongest correlation was observed for the subjective dimension (*r*_*s*_ =  − .51, 95% *CI* [−.661, −.324], *P* < .001). Avoidance-oriented coping style did not present any significant correlations with any of the studied dimensions of quality of life. Moderate and statistically significant correlations of quality of life and self-compassion (*r*_*s*_ = .46, 95% *CI* [.262–.621], *P* < .001) as well as ego-resiliency (*r*_*s*_ = .59, 95% *CI* [.420–.719], *P* < .001) were found. Moreover, both of resources studied were correlated with different styles of coping with stress. Self-compassion was correlated with emotion-oriented style (*r*_*s*_ =  − .71, 95% *CI* [−.809, −.582), *P* < .001) and ego-resiliency was correlated with task-oriented style (*r*_*s*_ = .56, 95% *CI* [.381–.697], *P* < .001), emotion-oriented (*r*_*s*_ =  − .24, 95% *CI* [−.445, −019], *P* = .034) as well as avoidance-oriented (*r*_*s*_ = .29, 95% *CI* [.072–.486], *P* = .010).

Table 3 presents the summary of hierarchical regression analysis. A 3-stage hierarchical multiple regression was conducted with Quality of life as the dependent variable. In the first stage 3 styles of coping with stress were entered (task-oriented, emotion-oriented, and avoidance-oriented). Self-compassion was entered at stage 2 and ego-resiliency at stage 3.

**Table 2:** Presents Spearman r correlation matrix.

		**1**	**2**	**3**	**4**	**5**	**6**	**7**	**8**	**9**	**10**
1 Task-oriented	Spearman’s rho	–									
	*p*-value	–									
	Upper 95% CI	–									
	Lower 95% CI	–									
2 Emotion-oriented	Spearman’s rho	0.020	–								
	*p*-value	0.863	–								
	Upper 95% CI	0.244	–								
	Lower 95% CI	−0.206	–								
3 Avoidance-oriented	Spearman’s rho	0.218	0.308**	–							
	*p*-value	0.059	0.007	–							
	Upper 95% CI	0.423	0.499	–							
	Lower 95% CI	−0.008	0.088	–							
4 Self-Compassion	Spearman’s rho	0.183	−0.714***	−0.108	–						
	*p*-value	0.114	<.001	0.352	–						
	Upper 95% CI	0.392	−0.582	0.120	–						
	Lower 95% CI	−0.045	−0.809	−0.326	–						
5 Ego-Resiliency	Spearman’s rho	0.559***	−0.243*	0.293*	0.413***	–					
	*p*-value	<.001	0.034	0.010	<.001	–					
	Upper 95% CI	0.697	−0.019	0.486	0.584	–					
	Lower 95% CI	0.381	−0.445	0.072	0.207	–					
6 Psychophysical	Spearman’s rho	0.242*	−0.406***	0.080	0.348**	0.451***	–				
	*p*-value	0.035	<.001	0.490	0.002	<.001	–				
	Upper 95% CI	0.443	−0.198	0.300	0.532	0.614	–				
	Lower 95% CI	0.018	−0.578	−0.148	0.133	0.251	–				
7 Psychosocial	Spearman’s rho	0.307**	−0.463***	0.081	0.400***	0.516***	0.642***	–			
	*p*-value	0.007	<.001	0.485	<.001	<.001	<.001	–			
	Upper 95% CI	0.498	−0.265	0.301	0.574	0.664	0.758	–			
	Lower 95% CI	0.087	−0.623	−0.147	0.192	0.329	0.487	–			
8 Subjective	Spearman’s rho	0.415***	−0.512***	−0.097	0.575***	0.616***	0.599***	0.648***	–		
	*p*-value	<.001	<.001	0.402	<.001	<.001	<.001	<.001	–		
	Upper 95% CI	0.586	−0.324	0.131	0.709	0.739	0.726	0.762	–		
	Lower 95% CI	0.210	−0.661	−0.316	0.402	0.453	0.431	0.495	–		
9 Metaphysical	Spearman’s rho	0.441***	−0.235*	0.085	0.267*	0.482***	0.462***	0.592***	0.586***	–	
	*p*-value	<.001	0.041	0.465	0.020	<.001	<.001	<.001	<.001	–	
	Upper 95% CI	0.606	−0.010	0.305	0.465	0.638	0.623	0.721	0.717	–	
	Lower 95% CI	0.239	−0.437	−0.143	0.045	0.288	0.264	0.422	0.415	–	
10 Quality of life	Spearman’s rho	0.390***	−0.490***	0.034	0.460***	0.590***	0.804***	0.866***	0.840***	0.767***	–
	*p*-value	<.001	<.001	0.769	<.001	<.001	<.001	<.001	<.001	<.001	–
	Upper 95% CI	0.566	−0.298	0.258	0.621	0.719	0.872	0.913	0.896	0.846	–
	Lower 95% CI	0.181	−0.644	−0.193	0.262	0.420	0.707	0.796	0.758	0.655	–

**Table 3:** Summarizes the hierarchical regression analysis for variables predicting quality of life.

Variable	*β*	*t*	*P*	*pr*^2^	*R*	*R^2^*	Δ*R*^2^
Step 1					.69	.47	.47
Task-oriented	.43	4.91^***^	<.001	.25		
Emotion-oriented	−.58	−6.41^***^	<.001	.36		
Avoidance-oriented	.14	1.47	.145	.03		
Step 2					.69	.47	0
Task-oriented	.42	4.48^***^	<.001	.22		
Emotion-oriented	−.55	−4.07^***^	<.001	.19		
Avoidance-oriented	.13	1.40	.167	.03		
Self-compassion	.04	.27	.791	.001		
Step 3					.75	.57	.10
Task-oriented	.22	2.18^*^	.032	.06		
Emotion-oriented	−.44	−3.48^***^	<.001	.14		
Avoidance-oriented	.02	.21	.837	<.001		
Self-compassion	−.05	−.36	.717	.002		
Ego-Resiliency	.42	3.86^***^	<.001	.18		

**Notes.**

* *p* < .05, ** *p* < .01, *** *p* < .001.

The hierarchical multiple regression revealed that at Stage one, styles of coping with stress contributed significantly to the regression model, *F* (3,72) = 21.44, *P* <  0.001 and accounted for 47% of the variation in Quality of Life. Adding Self-compassion was not significant for the model *F* (1,71) = .071, *P* = 0.791. Finally, adding Ego-resiliency explained additional 10% of the variation in Quality of Life and change in *R*^2^ was significant, *F* (1,70) = 14.91, *P* <  0.001. When all independent variables were included in the last stage, neither Avoidance-oriented style nor Self-compassion were significant predictors of Quality of Life. Together the five independent variables accounted for 57% of the variance in Quality of Life.

## Discussion

This research aimed to verify whether ego-resiliency and self-compassion played a significant role in coping strategies and quality of life amongst parents of children with autism disorder. The obtained results allow for a hypothesis that ego-resiliency and a task-oriented coping strategy were important indicators of the quality of life of parents of children with ASD. Self-compassion was also strongly correlated with all dimensions of quality of life; however, it was not a significant predictor. It can be assumed that of the two studied internal resources, ego-resiliency was stronger and, in a way, superior to self-compassion, which can be explained by the fact that ego-resiliency seems to be a broader psychological construct that encompasses more aspects, including issues related to self-compassion

The results showed a strong negative correlation between self-compassion and emotion-oriented coping (*r*_*s*_ = −.71). This finding supports hypothesis posed in previous research Johnson & ÓBrien (2013) indicating that self-compassion cannot be understood as “being full of self-pity”, fantasising about one’s own emotions; more importantly, paying attention to one’s emotional state does not mean dwelling on negative emotions, such as anger, sadness or anxiety. Also, these results are in line with other reports regarding negative relationships between self-compassion and emotion-focused coping strategies (Lloyd et al., 2019; Allen & Leary, 2010).

Similar results, although of weaker intensity, were obtained for ego-resiliency. Ego-resiliency showed significant positive correlations with task-oriented coping, as well as weak positive correlations with avoidance-oriented coping. These findings are in line with previous research (Vulpe & Dafinoiu, 2012) and indicate that ego-resiliency in itself is related to individual activity oriented towards solving present problems, as well as towards searching for substituting activities, especially looking for social contacts. Ego-resiliency can be associated with constant deficits in interpersonal relations, and, quite often, with a partial loss of social support experienced by the parents of individuals with autism once they have been diagnosed (Myers, Mackintosh & Goin-Kochel, 2009); thus, avoiding difficulties by engaging with new social contacts can be compensatory in nature, and ego-resiliency can play a resource-strengthening role in the search for new social contacts. Taking into account the level of engagement in the child’s disorder displayed by parents, who often give up their needs to be able to focus fully on their child, avoidance—in some cases—can be a form of self-care, and ego-resiliency can be an adaptive element. On the other hand, however, the correlations of ego-resiliency with both task-oriented coping and avoidance-oriented coping may indicate the need for flexible “switching” between these styles of coping, taking into account the changeability typical of the course of ASD, which would also be characteristic of resilience.

Both self-compassion and ego-resiliency showed the strongest correlations with the subjective dimension of quality of life. This is an important finding, as Straś-Romanowska, Oleszkowicz & Frąckowiak (2004) suggested that the subjective dimension underlined the individuality, independence, and freedom of an individual—three dimensions that are not always fulfilled by the parents of children with ASD who strongly believe that what is needed from them is full sacrifice for the well-being of the child and a relinquishment of their own needs (Pisula & Porębowicz-Dorsmann, 2017). Additionally, ego-resiliency was an important predictor of all of the studied dimensions of quality of life, apart from the psychophysical dimensions, with which self-compassion did not show any important correlations. However, this finding seems to be due to the fact that, from a theoretical point of view, a compassionate attitude towards oneself can be a subordinate component of ego-resiliency. Based on the correlation results, self-compassion was important only for the autonomous and interpersonal dimensions, which suggests that this construct supports mainly the issues of mindfulness and sensitivity to one’s own experiences in regard to oneself (non-judgemental and nonevaluative) and towards others (the aspect of common humanity discussed by Neff (2003), referring to the belief that life difficulties are a common experience for all people).

The research was carried out on a considerably small sample of 76 parents of children with autism spectrum disorder who were mostly women; therefore, when generalising the obtained results, caution is advised, and future research must be carried out on a larger, more diverse group. In addition, it would be worth considering the two-way relationship between children’s symptoms and parental stress: in the present research, child characteristics did not cause parental stress, but parental stress (caused by other factors) might have exacerbated children’s challenging behaviour. Although, the obtained results allow the formulation of new hypotheses on the importance of ego-resiliency and self-compassion for the functioning of parents of children with ASD. For their verification, an experimental study should be carried out that introduces an intervention in the form of workshops or other psychological activities aimed at the development of skills in the areas of these resources.

## Conclusions

Among the two studied internal resources, only ego-resiliency was an important predictor of the quality of life and its components. Thus, it must be considered whether the resilience is not a superior construct to self-compassion, as it was also significantly correlated with the studied variables, but compared with ego-resiliency, its influence was not as strong. The obtained results allow for a hypothesis that ego-resiliency and a task-oriented coping strategy were important indicators of the quality of life of parents of children with ASD. On the other hand, it was indicated that self-compassion is a skill involving taking a non-judgemental approach to one’s functioning rather than dwelling on negative emotions, as it presented negative relationship with emotion-oriented coping style. It must be highlighted that self-compassion is a construct that can be relatively easily developed (Hufnagle et al., 2018), hence it can be used as a potential protective factor in aiming interventions in parents of children with ASD (Torbet, Proeve & Roberts, 2019; Wong, Mak & Liao, 2016). Potential interventions including acceptance and commitment therapy (ACT) in this sample should be considered, as it was reported that it aims to develop self-compassion (Yadavaia, Hayes & Vilardaga, 2014) and enhance quality of life in parents of individuals with autism spectrum disorder (Corti et al., 2018; Hahs, Dixon & Paliliunas, 2019).

##  Supplemental Information

10.7717/peerj.11198/supp-1 Supplemental Information 1 Raw data.

10.7717/peerj.11198/supp-2 Supplemental Information 2 Self-Compassion Scale Short in Polish by Kocur (unpublished)

## Additional Information and Declarations

### Data Availability

The following information was supplied regarding data availability: Raw data are available in the Supplementary Files.
