# Supplementary material for: Self-compassion, ego-resiliency, coping with stress and the quality of life of parents of children with autism spectrum disorder
Source: PeerJ. 2021 May 3;9:e11198. doi: 10.7717/peerj.11198 (PMC8101449; doi:10.7717/peerj.11198)
Supplement: Supplemental Information 2 [file peerj-09-11198-s002.pdf]

Krótka skala współczującej postawy wobec samego siebie
(Raes, Pommier, Neff, Van Gucht, 2011)

Instrukcja: Proszę, odpowiedz na wszystkie pytania najlepiej, jak potrafisz. Jeśli nie jesteś pewien/pewna którejś odpowiedzi, proszę, wpisz najbardziej pasującą. To ważne, aby wypełnić wszystkie pytania. Nie ma złych, ani dobrych odpowiedzi, nie ma też podchwytliwych pytań.

1	2	3	4	5
zupełnie do mnie nie pasuje	słabo do mnie pasuje	trochę do mnie pasuje	raczej do mnie pasuje	bardzo do mnie pasuje

……	1.Kiedy nie podołam ważnej dla mnie rzeczy, przepełnia mnie poczucie własnej nieudolności.
……	2.Staram się wykazywać cierpliwość i zrozumienie wobec elementów mojej osobowości, których nie lubię.
……	3.Kiedy wydarza się coś bolesnego, staram się zachować wyważone spojrzenie na sytuacje
……	4.Kiedy jestem przygnębiony, mam tendencję do postrzegania innych ludzi jako prawdopodobnie szczęśliwszych ode mnie.
……	5.Staram się postrzegać porażki jako część ludzkiej natury.
……	6.Kiedy przechodzę przez bardzo trudny czas, okazuję sobie troskę i czułość, której potrzebuję
……	7.Kiedy coś mnie zdenerwuję, staram się zachować równowagę emocjonalną.
……	8.Kiedy nie powodzi mi się coś ważnego, czuję się samotny we własnej porażce.
……	9.Kiedy jestem przygnębiony, mam obsesję na punkcie wszystkiego co jest źle.
……	10.Kiedy czuję się w jakiś sposób nieudolny, staram się sobie przypomnieć, że to odczucie podziela większość ludzi.
……	11.Jestem krytyczny i potępiam moje wady i niedociągnięcia.
……	12.Jestem nietolerancyjny i niecierpliwy wobec pewnych elementów mojej osobowości.
